# GGN-GO: geometric graph networks for predicting protein function by multi-scale structure features

**DOI:** 10.1093/bib/bbae559

**Published:** 2024-11-01

**Authors:** Jia Mi, Han Wang, Jing Li, Jinghong Sun, Chang Li, Jing Wan, Yuan Zeng, Jingyang Gao

**Affiliations:** The College of Information Science and Technology, Beijing University of Chemical Technology, Beijing; The College of Information Science and Technology, Beijing University of Chemical Technology, Beijing; The College of Life Science and Technology, Beijing University of Chemical Technology, Beijing; The College of Information Science and Technology, Beijing University of Chemical Technology, Beijing; The College of Information Science and Technology, Beijing University of Chemical Technology, Beijing; The College of Information Science and Technology, Beijing University of Chemical Technology, Beijing; Microbial Resource and Big Data Center, Institute of Microbiology, Chinese Academy of Sciences; Chinese National Microbiology Data Center (NMDC); The College of Information Science and Technology, Beijing University of Chemical Technology, Beijing

**Keywords:** protein function prediction, geometric graph networks, multi-scale structural features, graph attention pooling, graph contrastive learning

## Abstract

Recent advances in high-throughput sequencing have led to an explosion of genomic and transcriptomic data, offering a wealth of protein sequence information. However, the functions of most proteins remain unannotated. Traditional experimental methods for annotation of protein functions are costly and time-consuming. Current deep learning methods typically rely on Graph Convolutional Networks to propagate features between protein residues. However, these methods fail to capture fine atomic-level geometric structural features and cannot directly compute or propagate structural features (such as distances, directions, and angles) when transmitting features, often simplifying them to scalars. Additionally, difficulties in capturing long-range dependencies limit the model’s ability to identify key nodes (residues). To address these challenges, we propose a geometric graph network (GGN-GO) for predicting protein function that enriches feature extraction by capturing multi-scale geometric structural features at the atomic and residue levels. We use a geometric vector perceptron to convert these features into vector representations and aggregate them with node features for better understanding and propagation in the network. Moreover, we introduce a graph attention pooling layer captures key node information by adaptively aggregating local functional motifs, while contrastive learning enhances graph representation discriminability through random noise and different views. The experimental results show that GGN-GO outperforms six comparative methods in tasks with the most labels for both experimentally validated and predicted protein structures. Furthermore, GGN-GO identifies functional residues corresponding to those experimentally confirmed, showcasing its interpretability and the ability to pinpoint key protein regions. The code and data are available at: https://github.com/MiJia-ID/GGN-GO

## Introduction

Proteins are central to the functioning of life, performing a multitude of functions. They catalyze or inhibit the transcription or translation of genes [1], transmit signals, and maintain cellular functions, thereby ensuring the normal operation of biological systems. Therefore, understanding the functions of proteins is crucial for disease mechanism research, drug target identification, and advancing precision medicine [2, 3]. High-throughput sequencing technologies have advanced rapidly. This has led to a significant increase in the number of protein sequences. However, the experimental identification of protein functions is time-consuming and costly, which cannot keep pace with the rapid growth of protein sequences. Therefore, it is urgent to develop efficient and accurate computational prediction methods [4].

Methods for prediction of protein functions fall into three categories: template-based methods, sequence-based methods, and structural-based methods [5]. Template-based methods, such as local comparison (Blast) [6] and domain comparison (FunFam) [7] rely on the assumption of homology, which states that if the sequences are similar, then the functions are also similar. However, in fact, similar sequences can have divergent functions, limiting these methods for distantly homologous proteins. Sequence-based methods extract features like evolutionary and interaction information via machine learning, which better handle distant homologous proteins. For instance, DeepGO-SE [8] and SPROF-GO [9] employ pre-trained language models for semantic reasoning and embedding extraction to predict protein functions. However, current machine learning primarily computes specific sequence features and faces challenges in directly extracting complex features.Sequence-based deep learning methods have tackled this issue. DeepGO [10] uses convolutional neural networks (CNN) and fully connected layers to learn complex features from protein sequences, while DeepGOPlus [11] uses deep CNNs to identify motifs. Recently, sequence feature-based methods have integrated ESM2 [12], ProtTrans [13], and other pre-trained protein language models as feature extraction modules [14–16]. These models use advanced feature extraction to capture complex sequence information, enhancing the accuracy of protein function prediction. Although sequence features are very important, structural features are equally critical, as protein sequences dictate structure, which in turn determines function [17]. Structural features, as a supplement to sequence features, are crucial for functional prediction.

Currently, most biological molecules, including proteins, are represented using Graph Convolutional Network (GCN) [18], which can represent protein structures as non-Euclidean graph structures with residues as nodes and edges connecting spatially adjacent residues. Each node receives feature information from neighboring nodes and aggregates this information at each layer to update the feature representations. DeepFRI [17] uses contact maps to construct protein networks and employs GCNs to propagate residue features; GAT-GO [14] uses inter-residue contact maps and protein sequence embeddings, aggregating node features through a graph attention network to improve the accuracy of protein function prediction; PFresGO [19] also combines protein sequence embeddings with predicted inter-residue contact maps, using an attention mechanism to capture this information and improving prediction accuracy by integrating the hierarchical structure of GO labels;HEAL [15] uses a hierarchical graph transformer networks to focus on key features during feature propagation and enhances the model’s representation capabilities through contrastive learning.

Despite advances in the application of GCNs, limitations remain: (i) existing protein function prediction methods fail to capture atomic-level geometric information, hindering understanding of active sites and molecular interactions, thus affecting accurate protein function prediction. (ii)Residue orientations, angles, and other structural features, cannot be directly propagated in GCNs [20] due to their design for undirected graphs, which simplifies these to scalars and affects prediction accuracy. (iii) Difficulties in capturing long-range dependencies restrict the model’s ability to identify key nodes (residues), which are vital for understanding protein function.

To address these challenges, we propose a method using a geometric graph network with multi-scale structural features (GGN-GO). Specifically,to obtain more comprehensive structural features, we capture multi-scale structural features in the protein, including angles and orientations between protein residues and atoms within the residues. To mitigate the loss of structural information during propagation within the geometric graph network, we introduce geometric vector perceptrons (GVPs) to convert these features into vector representations. After applying two linear transformations followed by a nonlinear transformation, we aggregate these representations with the node features, which have been converted to scalar values, to create a comprehensive graph representation. This representation is then input into the geometric graph network for propagation and computation.To improve the identification of key nodes, we introduce supernodes that interact with the protein graph nodes. We use graph attention pooling (GAP) to aggregate their representations. We also implement smooth perturbation of node features for normalization, enhancing robustness and generalization in identifying critical protein features.

To evaluate the effectiveness of GGN-GO in the prediction of protein function, we conducted comparative experiments between GGN-GO and BLAST, FunFam, DeepGO, DeepFRI, PFresGO, and HEAL. We train GGN-GO-PDB using protein structure datasets (PDBch) obtained from the Protein Data Bank (PDB). The results demonstrate that GGN-GO-PDB performs exceptionally well on PDBch, particularly outperforming other methods in the biological process (BP) tasks, which have the most functional labels. Subsequently, we further trained GGN-GO using predicted datasets (AFch) generated by deep learning methods. GGN-GO consistently outperformed all other methods on both the PDBch and AFch test sets, indicating its robust performance on newly discovered proteins as well. Furthermore, we used Grad-CAM to understand the key residues influencing GGN-GO’s decisions. The experimental results indicate that GGN-GO is capable of identifying crucial functional residue positions, thereby demonstrating its interpretability.

## Materials and methods

### An overview of GGN-GO

**Figure 1:**
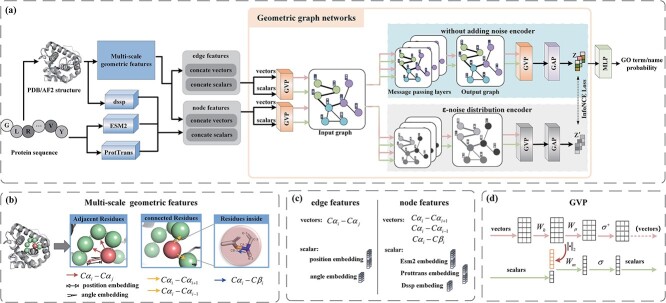
(a). Overview of the GGN-GO model. We extracted high-level sequence features using ESM2 and ProtTrans, and obtained secondary structure information with DSSP. Additionally, we extracted multi-scale geometric features from protein structures. These features were integrated into a graph representation with scalar and vector features by GVPs. Finally, the node and edge features were fed into the geometric graph network and optimized using a comparative learning strategy. (b) Details of Multiscale Geometric Features. This includes geometric vector features such as $C{\alpha _{i}}-C{\alpha _{j}}$ between $C\alpha $ atoms in neighboring residues, scalar features for angle and position embedding, geometric vector features $C{\alpha _{i - 1}} - C{\alpha _{i}}$and$C{\alpha _{i + 1}} - C{\alpha _{i}}$ between $C\alpha $ atoms in connected residues, and geometric vector features $C{\beta _{i}} - C{\alpha _{i}}$ between $C\alpha $ atoms inside the residue and $C\beta $ atoms in the side chain. (c) Details of edge features and node features. Edge features include vectors $C{\alpha _{i}} - C{\alpha _{j}}$, scalar positional embeddings, and angular embeddings. The node features consist of the vectors $C{\alpha _{i - 1}} - C{\alpha _{i}}$,$C{\alpha _{i + 1}} - C{\alpha _{i}}$ and $C{\beta _{i}} - C{\alpha _{i}}$, as well as the scalar features of ESM2, ProtTrans, and their respective feature embeddings. (d) Transformation process in GVP. The vectors and scalars input into the GVP undergo two linear and one nonlinear transformations for vectors, and one linear and one nonlinear transformation for scalars. The $\|.\|_{2}$ computation occurs after the vectors undergo the first linear transformation and are then integrated into the scalars.

To obtain richer features, we extracted both the sequence and structural features from each protein sample. For sequence features, we used the protein language models ESM2 [12] and ProtTrans [13]. For structural features, we used DSSP [21] to obtain secondary structure features and capture multi-scale geometric features of residues and within residues. These features were integrated into node and edge features, with undirected features as scalars and directed features as vectors. A graph representation was constructed using GVPs and processed in a geometric graph network, trained with supervised and graph comparison learning strategies. Details are shown in Fig. 1.

### Multi-scale geometric features

We first input the protein sequences into the ESM2 and ProtTrans models. Subsequently, based on the experimentally determined protein structures (PDB), we represented each protein as a graph $G(V,E)$. In this graph, $V$ denotes all the nodes (residues) of the protein, and $E$ represents the edges, which correspond to the distance relationships between the nodes. We then extracted multi-scale geometric features of the nodes and edges [22] (Fig. 1b) and integrated them with high-level sequence features to form the node and edge features of the protein (Fig. 1c). These features were subsequently input into the geometric graph network.

#### Node features

Node features contain vector and scalar features.

Vector features: (i) the central carbon atom of each residue $C\alpha $ in the protein serves as the starting point of the node vector, with the central carbon atoms of its two connected residues as vector endpoints ($C{\alpha _{i - 1}}-C{\alpha _{i}}$,$C{\alpha _{i + 1}} - C{\alpha _{i}}$). (ii) The second carbon atom $C\beta $ on the side chain to which the current residue $C\alpha $ is attached serves as the end point of another node vector ($C{\beta _{i}} - C{\alpha _{i}}$) [23].

Scalar features: (i) using DSSP to analyze the PDB structure, we obtained three types of features: one-hot encoded secondary structure profiles, peptide backbone torsion angles (PHI and PSI) represented by sine and cosine values, and solvent-accessible surface area. (ii) Node features were enhanced using pre-trained language models ESM2 and ProtTrans. These two models complement each other, enhancing the generalization ability to unknown sequences.

#### Edge features

Edge features also contain vector and scalar features.

Vector features: unit vectors from ${v_{i}}$ to ${v_{j}}$, where the direction between adjacent residues is $C{\alpha _{i}} - C{\alpha _{j}}$. Here, ${v_{j}}$ represents the current node, and ${v_{i}} - {v_{j}}$ represents its neighboring nodes.

Scalar features: (i) distance features encoded using the Gaussian Radial Basis Function [24] for distance ${\left \| {C{\alpha _{i}} - C{\alpha _{j}}} \right \|_{2}}$. (ii) Positional features encoded using the sine and cosine functions of the distances between ${v_{i}}$ and $ {v_{j}}$.

### Architecture of geometric graph networks

We use the node and edge features as inputs to the geometric graph network. The GVP processes these features through linear and nonlinear operations. Then, the GVP-based GNN updates the information on neighboring nodes and edges. Finally, a GAP layer learns the features of key nodes.

#### GVP

The GVP [25] is used to learn the vector and scalar features. For vector features, two linear transformations are followed by a nonlinear transformation. The first linear transformation maps features to a higher-dimensional space for better representation. The combination of the second linear and nonlinear transformations helps extract rotation-invariant information and allows the model to capture more complex feature representations through the introduction of nonlinear properties. For scalar features input to the GVP, the L2 norms of the vector features after the first linear transformation are concatenated with the input scalars to generate new scalar features. To further enhance the model’s representation and learning capabilities, these new scalar features undergo an additional linear and nonlinear transformation. The resulting features reduce dimensionality and complexity while retaining essential information, which helps to efficiently and accurately learn protein features.

The node and edge features were computed and propagated using two separate GVPs. The node and edge features computed by the GVPs are used to construct the input graph representation, which then serves as the input for the GVP-based GNN.


(1) \begin{gather*}\kern-.5pc V^{\prime} = {\sigma^ +} \left({\left\| {{{\mathrm{W}}_{\mu}}{{\mathrm{W}}_{h}}V} \right\|_{2}}\right) \odot \left({{\mathrm{W}}_{\mu}}{{\mathrm{W}}_{h}}V\right) \end{gather*}

(2) \begin{gather*} s^{\prime} = \sigma \left({{\mathrm{W}}_{m}}\left(concat\left({\left\| {{{\mathrm{W}}_{h}}V} \right\|_{2}},s\right)\right) + b\right) \end{gather*}

where $V\in{R^{{\text{v}}\times 3}}$ and $s\in{R^{\text{n}}}$ denote the vector and scalar features of the input GVP, respectively, $V^{\prime} \in{R^{\mu \times 3}}$ and $s^{\prime}\in{R^{\text{m}}}$ are the new features generated by the GVP.${\sigma ^{+}}$(.) and ${\sigma }$(.) are two different nonlinear transformation functions,${{\mathrm{W}}_{\mu }}$, ${{\mathrm{W}}_{h}} $ and ${{\mathrm{W}}_{m}}$ are three different linear weights, and $b$ is the bias term.

#### GVP based on GNN

We used graph neural networks to compute and propagate node information in protein graphs. Unlike previous methods [23] that propagate only undirected scalar features, our method incorporates feature directionality during propagation. We employed a GVP-based GNN for message transmission between nodes, using the graph representation of each protein as input. The propagation process is shown below:


(3) \begin{gather*}\kern-5pc {h_{m}}^{(j \to i)} = g\left(concat\left({h_{v}}^{(i)},{h_{e}}^{(j \to i)}\right)\right) \end{gather*}

(4) \begin{gather*} {h_{v}}^{(i)} = LayerNorm\left({h_{v}}^{(i)} + \frac{1}{{k^{\prime}}}\left(\sum\limits_{j:{e_{j \to i}} \in E} {{h_{m}}^{(j \to i)}} \right)\right) \end{gather*}

where the features of node $i$ and egde $(j-i)$ are represented as ${h_{v}}^{(i)}$ and ${h_{m}}^{(j \to i)}$, respectively, and $g$ represents the GVP module. ${h_{m}}^{(j \to i)}$ passed from node $j$ to node $i$, and $k^{\prime}$ is the dimensionality of the incoming features.

Since there was no need to learn the directionality of the features after the GVP-based GNN, and the GVP has already integrated directional information into the scalar features when calculating both the scalar and vector features (Fig. 1(d)), we added an additional GVP after it to incorporate directionality from the output graph into the scalar features for GAP. This simplifies the pooling operation’s complexity while maintaining feature expressiveness, improving network efficiency, and performance.

#### GAP

$n$ trainable important nodes as a query matrix, with each node’s feature dimension denoted by $d$. To learn more complex node relationships, we introduced the encoders $GC{N^{1}}$ and $GC{N^{2}}$ to obtain the key and value matrix. Next, we constructed the multi-head attention matrices using different parameters, namely ${\Gamma _{1}}$,...,${\Gamma _{H}}$, to further enhance the model’s focus on key nodes. Finally, we concatenated the multi-head attention matrices and used a fully connected layer to generate the important node matrix $A \in{R^{{\text{n}} \times{\text{d}}}}$.


(5) \begin{gather*} \Gamma = soft\max\left (\frac{{Q \cdot{K^{T}}}}{{\sqrt d}}\right)V \end{gather*}

(6) \begin{gather*}\kern-2.7pc K=GC{N^{1}}(G,E) \end{gather*}

(7) \begin{gather*} \kern-2.8pc\text{V}=GC{N^{2}}(G,E) \end{gather*}

(8) \begin{gather*}\kern-1.4pc {\text{A}} = {\text{MLP}}({\Gamma_{1}}, \ldots,{\Gamma_{H}}) \end{gather*}

where $G$ denotes the graph representation of each protein, $E$ represents the distance relationships between all nodes, and ${\text{MLP(.)}}$ is the multilayer perceptron used to transform the embedding of each significant node.

To further capture the key features and structural relationships of important nodes, we inputted the important node matrix into the GAP layer. Specifically, we defined a query vector $Q^{P} \in{R^{{\text{1}} \times{\text{d}}}}$ and use $GC{N^{1}}$ and $GC{N^{2}}$ to obtain the key matrices $K^{P} \in{R^{{\text{d}} \times{\text{d}}}}$ and the value matrix $V^{P} \in{R^{{\text{d}} \times{\text{d}}}}$, the formula is as follows:


(9) \begin{gather*} z = soft\max\left (\frac{{{Q^{P}} \cdot{{(A \cdot{K^{P}})}^{T}}}}{{\sqrt d}}\right)A \cdot{V^{P}} \end{gather*}

Finally, the output of the GAP, $z \in{R^{1 \times{\text{d}}}}$, is processed by the MLP, and the sigmoid activation function was applied to generate the prediction vector $\bar y \in{R^{1 \times C}}$.

### Graph contrastive learning

To better capture key information in graph data, we introduced graph comparison learning as a normalization strategy [26, 27]. Optimize similar graph representations to bring them closer and dissimilar ones to push them apart, effectively distinguishing structures and features in graphs. We added random noise ${\varepsilon _{v}}$ to the nodes of each graph representation, using the sign function sign($\cdot $), and compared the similarity between the original and perturbed graph representations:


(10) \begin{gather*} {h_{v}}^{\prime} = {h_{v}} + \left| {{\varepsilon_{v}}} \right| \cdot{\mathrm{sign}} ({h_{v}}) \end{gather*}

where ${h_{v}}^{\prime}$ is the perturbed graph representation.

To maximize similarity, we used the InfoNCE loss function [28]:


(11) \begin{gather*} {L_{reg}} = - \frac{1}{M}\sum\limits_{m = 1}^{M} {\log \frac{{{e^{{z_{m}} \circ z{^{\prime}_{m}}/\tau}}}}{{\sum_{m^{\prime} = 1}^{M} {{e^{{z_{m}} \circ z{^{\prime}_{m}}/\tau }}}}}} \end{gather*}

where $M$ is the number of samples in each batch, and $m$ is the $m$th protein sequence. Each sequence passes through the original GGN-GO to obtain a raw representation $z{_{m}}$, and through the perturbed GVP to get $z{^{\prime}_{m}}$. The temperature parameter $\tau $ is set to 0.5 [26]. The symbol $\circ $ computes the cosine similarity between vectors.

For the multi-label classification task, we use binary cross entropy as the supervised loss:


(12) \begin{gather*} {L_{\sup }} = - \frac{1}{{M \cdot C}}\sum\limits_{l = 1}^{C} {\sum\limits_{m = 1}^{M} {\left(y{\text{log}} (\bar y) + (1 - y){\text{log}} (1 - \bar y)\right)}} \end{gather*}

where $y$ and $\bar y$ denote the true probability and the predicted positive probability of the $l$th GO term for the $m$th sample, respectively, and $C$ represents the number of GO terms.The final loss of GGN-GO is the weighted sum of supervised and contrastive losses:


(13) \begin{gather*} L = {L_{\sup }} + {L_{reg}} \end{gather*}

### Implementation and settings for training

All GGN-GO experiments were performed on four NVIDIA RTX A6000 (48G) GPUs. Adam optimizer [29] was used with a learning rate of 0.0001 and a batch size of 64 for 100 epochs. The models were implemented using PyTorch and PyTorch Geometric [30]. Early stopping with five-epoch patience based on the validation set was applied to prevent overfitting [31].

## Datasets

We used the PDB dataset (PDBch) [32] and the SM dataset (SMch) [33] provided by DeepFRI (https://github.com/ flatironinstitute/DeepFRI), as well as the AF dataset (AFch) [15, 34] provided by HEAL (https://github.com/ ZhonghuiGu/HEAL). PDBch includes 36 641 protein structures from the PDB database, and SMch includes 244 775 protein structures from the SWISS-MODEL repository.

The processing of PDBch involves (i) obtaining all protein structures with contact maps in PDBch from the PDB; (ii) clustering by 95% sequence identity and selecting representative protein chains with at least one GO term; and (iii) dividing PDBch into training, validation, and test sets according to an 8:1:1 ratio.The protein chains and corresponding GO terms in PDBch were downloaded as ground truth values from the SIFTS [35] database, which collates information from the PDB [33] and UniProtKB [2] databases. After counting, the occurrences of GO terms in PDBch totaled 489 from Molecular Function (MF) terms, 1943 from BP terms, and 320 from Cellular Component (CC) terms.To further categorize all the GO terms in PDBch, we introduce a frequency score(IC), to calculate the occurrence frequency of each GO term in the PDBch training set. A higher IC indicates a lower frequency of occurrence and vice versa [32]:


(14) \begin{gather*} L = {L_{\sup }} + {L_{reg}} \end{gather*}

SMch data were collected by extracting protein structures with at least one GO term from PDBch, sourced from the SWISS-MODEL database, and clustering them at 95% identity. SMch was divided into training, validation, and test sets (8:1:1).

AFch was constructed by identifying proteins with low-frequency GO terms (IC <10) from the PDBch training set and retrieving 44,137 protein structures from the AlphaFold database [34]. The sequences were clustered with MMseqs at 25% sequence identity, resulting in a training set with 43,072 sequences and a test set with 567 sequences. Sequences in the AFch test set with more than 25% identity to those in the AFch and PDBch training sets were removed.

## Baseline methods

GGN-GO was compared with six methods, detailed in Supplementary Material S1. These include BLAST [36], an unsupervised method for functional annotation via sequence similarity; FunFams [7], a domain-based annotation method; DeepGO [10], a deep learning method for sequence and network features; DeepFRI [17], which combines sequence and structural information; PFresGO [19], which uses self-attention and cross-attention to capture protein functions; and HEAL [15], a hierarchical graph transformer method integrating sequence and structural information.

## Evaluation metrics

To compare the performance of different algorithms, we use CAFA evaluation criteria [37], including Fmax, AUPR, and Smin. Fmax finds the threshold for the highest F1 score, calculating precision and recall at various thresholds. AUPR measures the area under the precision-recall curve, providing a comprehensive assessment of the model’s performance at all thresholds. Smin weights GO terms with different ICs to evaluate the prediction of rare protein functions. Detailed calculations for each metric are provided in Supplementary Material (S2).

## Results

### Improvement of protein function prediction by GGN-GO

We evaluated the performance of GGN-GO on the PDBch and AFch test sets by comparing it to Blast, FunFams, DeepGO, DeepFRI, PFresGO and HEAL. DeepGO, DeepFRI, and PFresGO were trained on the PDBch and SMch training sets, and HEAL was trained on the PDBch, PDBch+SMch, and PDBch+AFch training sets. For comparison, we trained GGN-GO on the PDBch, PDBch+SMch, and PDBch+AFch training sets. The resulting models were named GGN-GO-PDB, GGN-GO-SM, and GGN-GO. The performance of GGN-GO in the three gene ontology domains (MF, BP, CC) was assessed using three evaluation metrics (AUPR, Fmax, Smin), as shown in Table 1.

**Table 1:** AUPR, Fmax, and Smin values of different methods on the PDBch test set, with the highest Fmax and AUPR and the lowest Smin highlighted in bold

**Method**	**Training set**	**AUPR ($\uparrow $)**			**Fmax ($\uparrow $)**			**Smin ($\downarrow $)**		
		**MF**	**BP**	**CC**	**MF**	**BP**	**CC**	**MF**	**BP**	**CC**
Blast	–	0.136	0.067	0.097	0.328	0.336	0.448	0.632	0.651	0.628
FunFams	–	0.367	0.260	0.288	0.572	0.500	0.672	0.531	0.579	0.503
DeepGO	PDBch+SMch	0.391	0.182	0.263	0.577	0.493	0.549	0.472	0.577	0.550
DeepFRI	PDBch+SMch	0.495	0.261	0.274	0.625	0.540	0.613	0.437	0.543	0.527
PFresGO	PDBch+SMch	0.602	0.293	0.361	0.692	0.568	0.674	0.417	0.535	0.498
HEAL-PDB	PDBch	0.571	0.259	0.342	0.691	0.565	0.655	0.401	0.540	0.501
HEAL-SW	PDBch+SMch	0.653	0.308	0.432	0.711	0.581	0.654	0.366	0.509	0.489
HEAL	PDBch+AFch	0.691	0.337	0.467	0.747	0.595	0.687	**0.342**	0.509	**0.458**
GGN-GO-PDB	PDBch	0.601	0.291	0.347	0.698	0.643	0.657	0.400	0.546	0.510
GGN-GO-SM	PDBch+SMch	0.684	0.339	0.438	0.718	0.659	0.657	0.368	0.513	0.498
GGN-GO	PDBch+AFch	**0.708**	**0.418**	**0.481**	**0.758**	**0.677**	**0.699**	0.348	**0.500**	0.463

GGN-GO-PDB achieved AUPR scores of 0.601, 0.291, and 0.347, Fmax scores of 0.698, 0.643, and 0.657, and Smin scores of 0.400, 0.546, and 0.510 for MF, BP, and CC tasks. Trained on PDBch, GGN-GO-PDB outperforms Blast, FunFams, DeepGO, DeepFRI, and PFresGO. It also shows superior AUPR and Fmax scores compared to HEAL-PDB, especially in BP, with similar Smin scores.

GGN-GO-SM achieved the following scores for MF, BP, and CC tasks: AUPR scores of 0.684, 0.339, and 0.438; Fmax scores of 0.718, 0.659, and 0.657; and Smin scores of 0.368, 0.513, and 0.498, respectively. GGN-GO-SM outperforms Blast and FunFams across all domains and competes well with DeepGO, DeepFRI, PFresGO, and HEAL-SMch trained on PDBch + SMch.

GGN-GO achieved AUPR scores of 0.708, 0.418, and 0.481, Fmax scores of 0.758, 0.677, and 0.699, and Smin scores of 0.348, 0.500, and 0.463 for MF, BP, and CC tasks. Trained on PDBch+AFch, GGN-GO outperformed all comparison methods in BP, the task with the most function labels.

By using different random seeds, we conducted 20 training sessions and performed paired-sample t-tests on the AUPR scores of the models on the test dataset for each training session. The $P$-values for comparisons between GGN-GO or GGN-GO-PDB models and other baselines were below 0.05 at $\alpha = 0.05$, indicating our models significantly outperform the baselines.

### Ablation study of components

To evaluate the contribution of different components in GGN-GO, we performed ablation experiments using the PDBch and AFch test sets. The four ablated models are as follows: (i) GGN-GO w/o LLM: Removes feature embeddings from ESM2 and ProtTrans. (ii) GGN-GO w/o GVP: Remove vector processing in GVP, turning it into a scalar-only perceptual machine. (iii) GGN-GO w/o GAP: Removes GAP. (IV) GGN-GO w/o CL: Removes comparative learning optimization. The results are shown in Table 2.

**Table 2:** AUPR, Fmax, and Smin values of different methods on the PDBch test set, with the highest Fmax and AUPR and the lowest Smin highlighted in bold

**Method**	**AUPR ($\uparrow $)**			**Fmax ($\uparrow $)**			**Smin ($\downarrow $)**		
	**MF**	**BP**	**CC**	**MF**	**BP**	**CC**	**MF**	**BP**	**CC**
GGN-GO	**0.708**	**0.418**	**0.481**	**0.758**	**0.677**	**0.683**	**0.355**	**0.500**	**0.473**
GGN-GO w/o LLM	0.315	0.268	0.284	0.489	0.461	0.583	0.571	0.621	0.568
GGN-GO w/o GVP	0.638	0.311	0.388	0.672	0.571	0.643	0.421	0.547	0.512
GGN-GO w/o GAP	0.584	0.307	0.371	0.660	0.569	0.629	0.453	0.567	0.527
GGN-GO w/o CL	0.658	0.383	0.462	0.713	0.659	0.668	0.382	0.517	0.493

Experimental findings indicate that removal of ESM2 and ProtTrans significantly affects AUPR scores (0.315, 0.268, 0.284), Fmax (0.489, 0.461, 0.583) and Smin (0.571, 0.621, 0.568) on MF, BP, and CC tasks, underscoring the pivotal role of protein language models in advance of sequence analysis. Additionally, excluding GVP reduces AUPR (0.638, 0.311, 0.388) and Fmax (0.672, 0.571, 0.643), and raises Smin (0.421, 0.547, 0.512), particularly in BP, implying that integrating scalar and vector features enhances the model’s grasp of intricate protein attributes. Removing GAP yields poorer results than GVP removal (AUPR: 0.584, 0.307, 0.371; Fmax: 0.660, 0.569, 0.629; Smin: 0.453, 0.567, 0.527), indicating GAP’s capture of pivotal nodes. Without contrastive learning optimization, the performance of the model decreases slightly (AUPR: 0.658, 0.383, 0.462; Fmax: 0.713, 0.659, 0.668; Smin: 0.382, 0.517, 0.493), which highlights its role in the identification of critical features amid noisy data.

### Ablation study of multi-scale features

Since our study focuses on multidimensional geometric features such as vectors and angles of nodes and edges, we performed feature ablation experiments using PDBch and AFch test sets to evaluate the contribution of different geometric dimensions to the prediction results of GGN-GO. The three ablation experiments are as follows: (i) GGN-GO w/o ProtTrans: removes ProtTrans feature embedding. (ii) GGN-GO w/o ESM2: removes ESM2 feature embeddings. (iii) GGN-GO w/o DSSP + DA: removes the secondary structure of the protein and the features of the dihedral angle extracted by DSSP.

The results indicate that without ProtTrans, GGN-GO achieves AUPR scores of 0.641, 0.341, and 0.427, Fmax scores of 0.672, 0.623, and 0.612, and Smin scores of 0.43, 0.531, and 0.501 for the MF, BP and CC tasks (Table 3). This underscores the importance of the ProtTrans features. Removing ESM2 yields AUPR scores of 0.647, 0.363, and 0.439, Fmax scores of 0.661, 0.628, and 0.608, and Smin scores of 0.421, 0.537, and 0.499 for the same tasks, similar to performance after removing ProtTrans. Both models improve protein function prediction by providing advanced sequence information. Removing DSSP and dihedral angle features (20 dimensions) compared to ProtTrans and ESM2 (1024 and 1280 dimensions) results in AUPR scores of 0.697, 0.383, and 0.467, Fmax scores of 0.734, 0.659, and 0.661, and Smin scores of 0.368, 0.512, and 0.479 for the MF, BP, and CC tasks, demonstrating strong performance and the importance of structural features.

**Table 3:** AUPR, Fmax, and Smin values of different methods on the PDBch test set, with the highest Fmax and AUPR and the lowest Smin highlighted in bold

**Method**	**AUPR ($\uparrow $)**			**Fmax ($\uparrow $)**			**Smin ($\downarrow $)**		
	**MF**	**BP**	**CC**	**MF**	**BP**	**CC**	**MF**	**BP**	**CC**
GGN-GO	**0.708**	**0.418**	**0.481**	**0.758**	**0.677**	**0.683**	**0.355**	**0.505**	**0.473**
GGN-GO w/o ProtTrans	0.641	0.341	0.427	0.672	0.623	0.612	0.430	0.531	0.501
GGN-GO w/o ESM2	0.647	0.363	0.439	0.661	0.628	0.608	0.421	0.537	0.499
GGN-GO w/o dssp+DA	0.697	0.383	0.467	0.734	0.659	0.661	0.368	0.512	0.479

### Generalizability of GGN-GO

**Figure 2:**
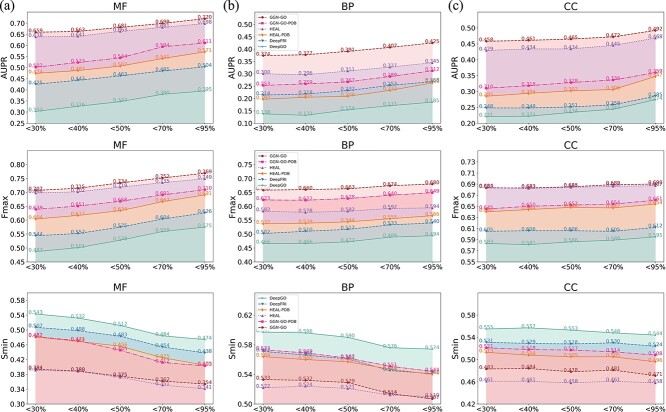
(a) AUPR, (b) Fmax and (c) Smin of different methods on different homology data from the PDBch test set.

To assess GGN-GO’s generalizability, we evaluated its performance on PDBch test data with homology thresholds of 30%, 40%, 50%, 70%, and 95% and compared GGN-GO, GGN-GO-PDB, DeepFRI, and DeepGO (Fig. 2). The results show that GGN-GO-PDB and DeepFRI outperform DeepGO, indicating that structural information improves prediction accuracy. GGN-GO outperforms DeepFRI and DeepGO at all thresholds and achieves results comparable to HEAL, excelling in BP tasks. Lower homology boosts performance across methods, but GGN-GO achieves similar results on lower homology data as others do on higher homology, suggesting multi-scale structure features enhance its learning of structure-function relationships. See Supplementary Material (S3.1) for details.

### Performance of GGN-GO on the Language Model predicted structures

**Figure 3:**
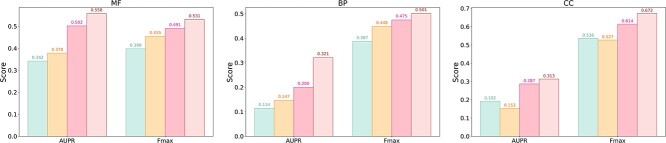
AUPR and Fmax of MF, BP, and CC obtained by different methods on the AFch test set.

A practical scenario for GGN-GO is predicting the functions of proteins lacking experimental structures or similar sequences. We benchmark GGN-GO against methods using sequence and homology data on the AFch test set, comparing it with DeepFRI, GGN-GO-PDB, and HEAL. Figure 3 shows that while DeepFRI trains on homology-modeled AFch sequences, GGN-GO-PDB, trained only on PDB structures, achieves comparable results. HEAL trains on AFch data with predicted structures. GGN-GO models trained on AFch data outperformed others, with higher Fmax scores (0.531, 0.501, 0.672) and AUPR scores (0.558, 0.321, 0.313) (Supplementary Material S3.2). These results highlight GGN-GO’s effectiveness in predicting structures without experimental resolution.

### Performance of GGN-GO for different specific GO terms

To evaluate GGN-GO performance across GO terms of varying specificity, we computed the information content (IC) distribution of GO terms in the PDBch and PDBch+AFch training sets (Supplementary Material Figs 1 and 2). The proteins in the PDBch test set were classified based on IC into three groups: low ($IC < 5$), moderate ($5 < IC < 10$), and high- specificity ($IC> 10$). In this experiment, the low specificity terms were found to be relatively common, while the moderate specificity terms exhibited intermediate specificity. High-specificity terms are found in only a few proteins, but they usually have significant biological importance and can be hard to predict. We used AUPR as the main metric to compare GGN-GO, GGN-GO-PDB, HEAL, HEAL-PDB, DeepFRI, and DeepGO. GGN-GO achieved AUPRs of 0.803, 0.529, and 0.401 for low, moderate, and high specificity terms, respectively. Its high-specificity performance significantly outperformed other methods (Supplementary Material Fig. 3). GGN-GO showed clear improvements in predicting highly specific functions.

### Performance of GGN-GO in recognizing key residues

To clearly show the contribution of each residue to the prediction, we applied a gradient-weighted class activation map (Grad-CAM) [38]. This approach is widely used in the visual interpretation of CNN classifiers to identify regions that contribute the most to the final decision by highlighting gradient changes. In analyzing a single protein sample, we selected the output after the GVP-based GNN and GVP as the feature map $F \in{R^{L \times D}}$, where $L$ is the protein sequence length and $D$ is the hidden dimension. We use the derivative of the protein function ${y^{l}}$ with respect to ${F_{i,j}}$ as the gradient weight $W_{i,j}^{l}$.


(15) \begin{gather*} W_{i,j}^{l} = \frac{{\partial{y^{l}}}}{{\partial{F_{i,j}}}} \end{gather*}

The contribution score of each residue can be obtained by the weighted summation of ${F_{i,j}}$ with $W_{i,j}^{l}$:


(16) \begin{gather*} CAM_{i}^{l} = {\mathrm{ReLU}} \left( {\frac{{\sum\nolimits_{j = 1}^{D} {W_{i,j}^{l}} {F_{i,j}}}}{D}} \right) \end{gather*}

**Figure 4:**
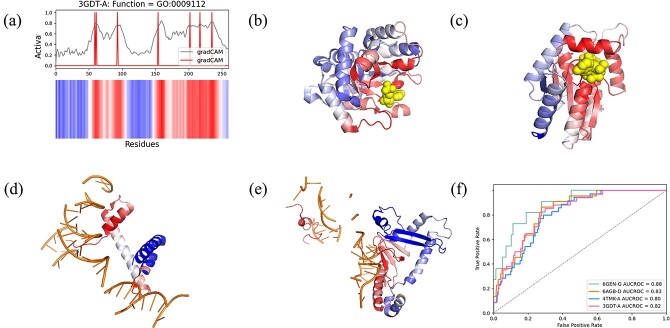
Visualization of GGN-GO’s predicted attention for four protein samples using Grad-CAM. (a) The weighted summation of visualization weights and feature maps is shown as the final predicted contribution, with red indicating a large contribution and blue a small contribution. (b)–(e) Mapping the visualization results onto the 3D structures of the corresponding proteins. (f) ROC curves and AUC scores for different samples.

Figure 4 A shows the $CAM_{i}^{l}$ heatmap for the GO:0009112 function numbered 3GDT-A.

To verify the regions contributing most to GGN-GO predictions, we obtained protein-ligand binding site data from the BioLip database [39]. Binding sites in BioLip are based on PDB experimental structures. A residue is considered bound to the nucleic acid if the van der Waals radii sum of the closest atoms between the residue and ligand is less than 0.5 Å. Residues closer to the ligand likely contribute more to the predictions for ligand-binding proteins [23].

For the BP task, we selected 3GDT, involved in nucleobase metabolism (GO:0009112). Its heat map with the binding site of 6-AZA-UMP is shown in Fig. 4(b), and residues close to the ligand contribute more to the prediction. Next, we selected 4TMK, involved in nucleoside triphosphate metabolism (GO:0009141), as shown in Fig. 4(c). Residues around the inhibitor TP5A significantly contributed to the heat map.

For the MF task, we selected the DNA polymerase 6GEN (GO:0003677) with DNA-binding function. The heatmap projected onto the protein structure showed strong signals in the DNA-binding region (Fig. 4d). We also selected RNA polymerase 6AGB (GO: 0003723) with RNA binding function and observed strong signals in the RNA-binding region (Fig. 4e).

Furthermore, we used ROC curves to compare the performance in identifying significant functional residues. The ROC curves show the relationship between the true positive rate (sensitivity) and the false positive rate (1-specificity), quantified by the area under the curve (AUC). A larger AUC implies higher accuracy in the prediction at the residue level. Fig. 4(f) shows the ROC curves and AUC values: 6GEN-G (0.88), 6AGB-D (0.83), 4TMK-A (0.80), and 3GDT-A (0.82), all indicating high predictive precision.

## Discussion

Currently, research on protein function prediction increasingly emphasizes the use of geometric structural information to understand protein functions. In this study, we propose the GGN-GO, which integrates multi-scale geometric structural features, including direction vectors between residues and their internal atoms, dihedrals, and secondary structures. These features undergo two linear and one nonlinear calculations using GVP, transforming them into vectors and scalars to construct a geometric graph representation. After propagation through a geometric graph network, key nodes are identified using GAP for decision-making. Additionally, contrastive learning is utilized for regularization. GGN-GO significantly improves prediction accuracy on large-scale functional tag sets and overall outperforms the current state-of-the-art model, HEAL, particularly demonstrating a significant enhancement in performance on BP tasks. This indicates that GGN-GO effectively propagates geometric structural features, playing a crucial role in functional prediction.

Experimental results show that as the volume of data and scale of labels increase, GGN-GO’s performance significantly improves, particularly excelling in large-scale the BP task and surpassing the state-of-the-art model HEAL, demonstrating adaptability to complex structural information. Ablation experiments confirm the contribution of various components and features to performance; generalization experiments reveal strong performance on low sequence similarity proteins; experiments on AFch validate its excellent performance on unknown structure proteins; experiments on different specificity data show significant improvements in prediction accuracy for high-frequency GO terms; and key residue identification confirms GGN-GO’s interpretability.

GGN-GO uses geometric structural features to understand protein functional regions, showing potential for predicting protein interaction sites, offering value in drug target identification and structural biology. In practice, GGN-GO demonstrates interpretability and reliability for unannotated proteins, advancing protein design in drug discovery and synthetic biology.

While GGN-GO performs well, it requires experimentally determined or predicted protein structures, which adds a step compared to sequence-based methods, increasing the complexity. This reliance can be limiting when structural data are unavailable or difficult to obtain. However, with advances in AlphaFold3 [40], the quality of structure prediction has significantly improved, partially alleviating this limitation. In the future, we hope to use protein structure prediction models and structural information for annotating unknown protein sequences.

**Key Points**

- GGN-GO enriches feature extraction by capturing multi-scale geometric structural features at the atomic and residue levels.
- GGN-GO uses GVPs to convert these features into vector representations and aggregates them with node features for better understanding and propagation within the geometric graph network.
- GGN-GO introduces supernodes and graph attention pooling to enhance the identification of key nodes.

## Supplementary Material

Supplementary_material_bbae559

Supplementary_material_bbae559

## Data Availability

The code and data are available at: https://github.com/MiJia-ID/GGN-GO.
