# Supplementary material for: GGN-GO: geometric graph networks for predicting protein function by multi-scale structure features
Source: Brief Bioinform. 2024 Nov 1;25(6):bbae559. doi: 10.1093/bib/bbae559 (PMC11530295; doi:10.1093/bib/bbae559)
Supplement: Supplementary_material_bbae559 [file supplementary_material_bbae559.pdf]

1 Baseline

GGN-GO was compared with six methods: BLAST, FunFams, DeepGO, DeepFRI, PFresGO, and HEAL.

(1)BLAST: An unsupervised learning method that annotates functions based on sequence similarity. The comparative experiment referenced the BLAST settings in CAFA1, with the default E value set to 1e-3. The functional annotations of the training set sequences were applied to the test set sequences, using sequence identity scores as prediction scores.

(2)FunFams: An unsupervised method that uses domain-based protein function annotation. For each protein sequence, the HMMER3 tool searches within CATH FunFams, and the functional category with the highest HMM score is annotated to the test sequence. The prediction score is the frequency of the occurrence of the functional category.

(3)DeepGO: A deep learning-based protein function prediction method that learns the deep features of sequences and topological features of the network to predict protein functions. This method improves prediction accuracy and coverage, especially when handling large-scale protein data.

(4)DeepFRI: Combine protein sequence and structure information by extracting sequence features through a pre-trained long-short-term memory (LSTM) model and creating a contact map between amino acids based on protein structure. The LSTM outputs and the constructed contact map are jointly input into a Graph Convolutional Network (GCN) to predict protein functions.

(5)PFresGO: Integrates the hierarchical structure of the Gene Ontology (GO) graph, using self-attention operations to capture the interrelationships of GO terms and cross-attention operations to embed protein representations and GO into a common latent space, identifying global protein sequence patterns and local functional residues.

(6)HEAL: Using protein sequence and structure information to capture

super nodes with a hierarchical graph transformer, inferring long-range dependencies between atoms, and combining distance maps between protein atoms to jointly predict protein functions.

2 Evaluation metrics

2.1 Fmax

$$Fmax = \max_t \left\{ \frac{2AvgPr(t) * AvgRc(t)}{AvgPr(t) + AvgRc(t)} \right\} \tag{1}$$

$$AvgPr(t) = \frac{1}{m(t)} * \sum_{i=1}^{m(t)} pr_i(t) \tag{2}$$

$$AvgRc(t) = \frac{1}{n} * \sum_{i=1}^{n} rc_i(t) \tag{3}$$

$Fmax$ is the maximum F1 score (Eq.1) obtained from all threshold calculations. The average precision score $AvgPr(t)$ and the average recall score $AvgRc(t)$ are shown in eq2 and eq3, respectively, for a given threshold t, where $pr_i(t)$ and $rc_i(t)$ denote the precision and recall scores for each protein. The threshold ttt takes values from 0 to 1 in steps of 0.01. Here, n is the number of proteins in the test set and $m(t)$ is the number of proteins with at least one function.

2.2 AUPR

$$macro - AUPR = \int Avg\overset{'}{\Pr}(t) * d\left(AvgRc'(t)\right) \tag{4}$$

$$AvgPr'(t) = \frac{1}{f(t)} \sum_{j=1}^{n} pr'_j(t) \tag{5}$$

$$AvgRc'(t) = \frac{1}{f(t)} \sum_{j=1}^{n} rc'_j(t) \tag{6}$$

We use macro-AUPR to evaluate model performance. Macro-AUPR refers to the area under the $AvgPr'(t)$ and $AvgRc'(t)$ curves (eq4. This is different from $AvgPr(t)$ and $AvgRc(t)$, which denote the average precision per protein and the recall at the threshold t. $AvgPr'(t)$ and $AvgRc'(t)$denote the average precision and recall for each protein function at threshold t (eq5, eq6).

2.3 Smin

$$Smin = \min_t \sqrt{ru(t)^2 + mi(t)^2} \tag{7}$$

$$ru(t) = \frac{1}{n} \sum_{i=1}^{n} \sum_{c \in T_i - P_i(t)} IC(l) \tag{8}$$

$$mi(t) = \frac{1}{n} \sum_{i=1}^{n} \sum_{c \in P_i - T_i(t)} IC(l) \tag{9}$$

$$IC\left(l\right) = -\log_2\left(P\left(l\right)\right) \tag{10}$$

$Smin$ is used to compute the minimum semantic distance (eq(7)) between the true and predicted annotations at different thresholds ttt. The performance of the model in the prediction of annotations is evaluated by combining residual uncertainty and error information. $ru(t)^2$ and $mi(t)^2$ denote the

average residual uncertainty and the average error information, respectively. The formulas for $ru(t)$ and $mi(t)$ are shown in eq.(8) and eq.(9), respectively, where T_i is the true annotation of protein i, $IC(l)$ quantifies the information content of the function l, and $P(l)$ is the probability of occurrence of function l.

3 Result

3.1 Generalizability of GGN-GO

Table 1: Evaluation metrics for GGN-GO and other competing methods on PDBch's test set at 5 homology thresholds for PDBch's test set

	AUPR (mf)				
Model	<30%	<40%	<50%	<70%	<95%
DeepGO	0.303	0.326	0.347	0.38	0.395
DeepFRI	0.425	0.443	0.463	0.485	0.504
HEAL-PDB	0.474	0.487	0.507	0.541	0.571
HEAL	0.638	0.641	0.663	0.681	0.698
GGN-GO-PDB	0.502	0.523	0.544	0.594	0.611
GGN-GO	0.659	0.663	0.681	0.699	0.72

	AUPR (bp)				
Model	<30%	<40%	<50%	<70%	<95%
DeepGO	0.138	0.132	0.154	0.171	0.185
DeepFRI	0.214	0.218	0.232	0.253	0.268
HEAL-PDB	0.197	0.205	0.209	0.233	0.263
HEAL	0.3	0.296	0.311	0.327	0.345
GGN-GO-PDB	0.253	0.259	0.267	0.289	0.312
GGN-GO	0.374	0.377	0.391	0.407	0.425

Model	AUPR(cc)				
	<30%	<40%	<50%	<70%	<95%
DeepGO	0.221	0.222	0.234	0.244	0.274
DeepFRI	0.248	0.248	0.251	0.258	0.285
HEAL-PDB	0.285	0.294	0.302	0.306	0.347
HEAL	0.429	0.434	0.434	0.445	0.468
GGN-GO-PDB	0.31	0.318	0.328	0.336	0.359
GGN-GO	0.458	0.461	0.465	0.472	0.492

Model	Fmax(mf)				
	<30%	<40%	<50%	<70%	<95%
DeepGO	0.487	0.501	0.528	0.559	0.575
DeepFRI	0.544	0.552	0.575	0.604	0.626
HEAL-PDB	0.604	0.617	0.634	0.667	0.691
HEAL	0.698	0.702	0.719	0.735	0.749
GGN-GO-PDB	0.639	0.651	0.668	0.691	0.71
GGN-GO	0.707	0.715	0.734	0.752	0.769

Model	Fmax(bp)				
	<30%	<40%	<50%	<70%	<95%
DeepGO	0.466	0.466	0.472	0.489	0.494
DeepFRI	0.502	0.51	0.517	0.533	0.54
HEAL-PDB	0.537	0.539	0.544	0.555	0.566
HEAL	0.582	0.578	0.582	0.592	0.594
GGN-GO-PDB	0.623	0.622	0.628	0.64	0.649
GGN-GO	0.658	0.66	0.663	0.674	0.68

Model	Fmax(cc)				
	<30%	<40%	<50%	<70%	<95%
DeepGO	0.583	0.581	0.586	0.589	0.595
DeepFRI	0.605	0.606	0.606	0.605	0.612
HEAL-PDB	0.64	0.644	0.648	0.647	0.654
HEAL	0.684	0.682	0.684	0.686	0.687
GGN-GO-PDB	0.645	0.65	0.652	0.654	0.661
GGN-GO	0.676	0.682	0.679	0.68	0.685

	Smin(mf)				
Model	<30%	<40%	<50%	<70%	<95%
DeepGO	0.543	0.532	0.512	0.484	0.474
DeepFRI	0.507	0.498	0.483	0.454	0.438
HEAL-PDB	0.481	0.469	0.456	0.425	0.405
HEAL	0.391	0.388	0.372	0.351	0.341
GGN-GO-PDB	0.482	0.471	0.445	0.412	0.403
GGN-GO	0.394	0.389	0.375	0.362	0.354

	Smin(bp)				
Model	<30%	<40%	<50%	<70%	<95%
DeepGO	0.597	0.596	0.59	0.576	0.574
DeepFRI	0.571	0.566	0.561	0.545	0.541
HEAL-PDB	0.564	0.56	0.557	0.547	0.54
HEAL	0.522	0.524	0.521	0.512	0.51
GGN-GO-PDB	0.573	0.569	0.562	0.551	0.542
GGN-GO	0.533	0.532	0.529	0.514	0.507

	Smin(cc)				
Model	<30%	<40%	<50%	<70%	<95%
DeepGO	0.555	0.557	0.553	0.548	0.544
DeepFRI	0.531	0.529	0.528	0.53	0.524
HEAL-PDB	0.513	0.508	0.505	0.505	0.496
HEAL	0.461	0.461	0.458	0.461	0.458
GGN-GO-PDB	0.521	0.518	0.517	0.514	0.508
GGN-GO	0.483	0.484	0.478	0.481	0.471

3.2 Performance of GGN-GO for predicting protein function with missing structure

Table 2: Model performance on AFch with respect to Fmax and macro-AUPR

	AUPR			Fmax		
Method	MF	BP	CC	MF	BP	CC
DeepFRI	0.342	0.114	0.192	0.398	0.387	0.536
GGN-GO-PDB	0.378	0.147	0.153	0.455	0.448	0.527
HEAL	0.502	0.2	0.287	0.491	0.475	0.614
GGN-GO	0.558	0.321	0.313	0.531	0.501	0.672

3.3 Performance of GGN-GO for different specific GO terms

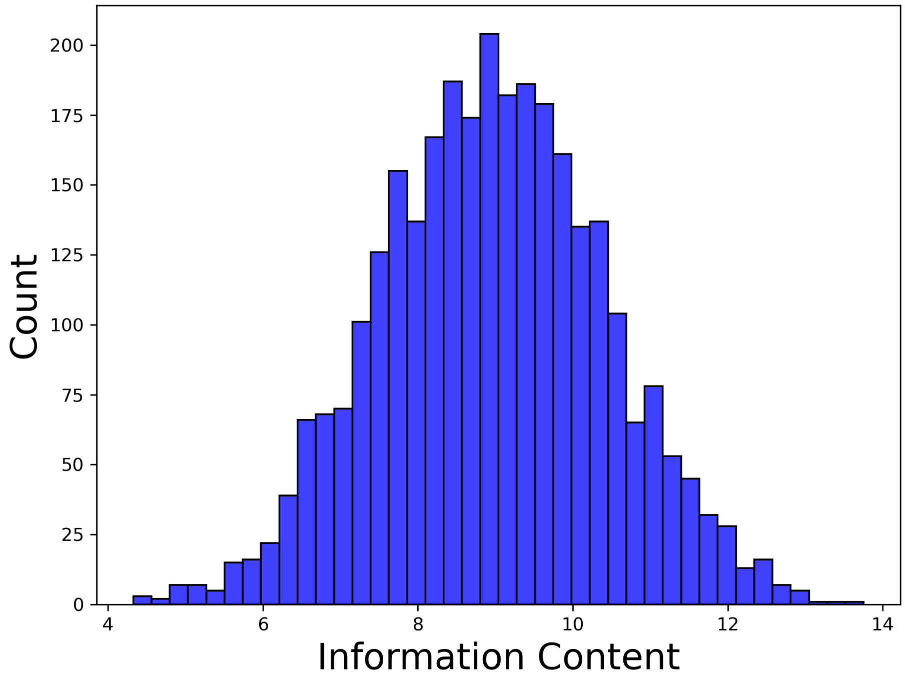

Figure 1: Distribution of Information Content (IC) for protein functions in the PDBch training set.

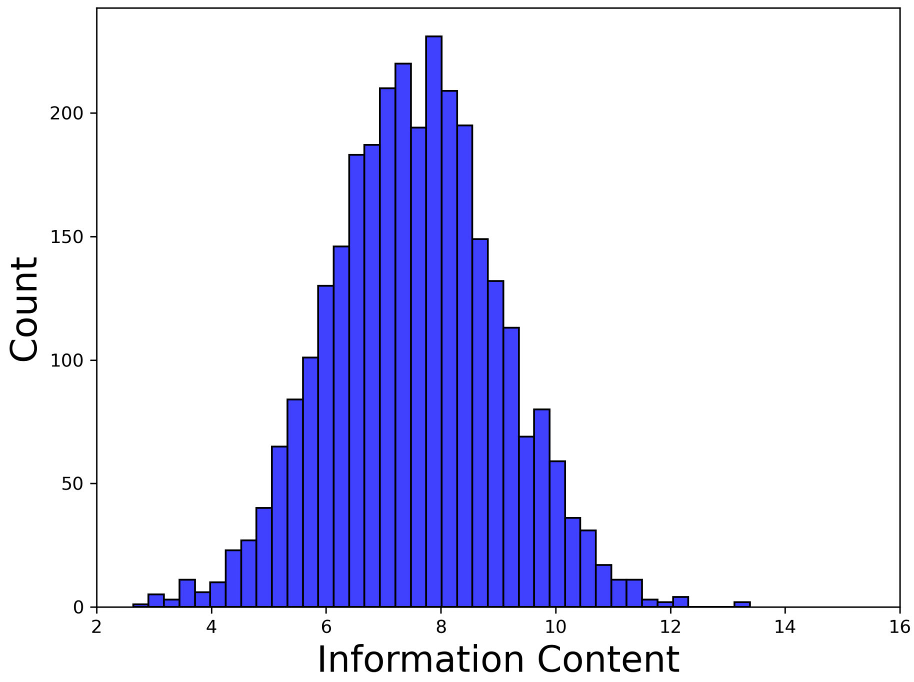

Figure 2: Distribution of Information Content (IC) for protein functions in the combination of PDBch training set and AFch training set.

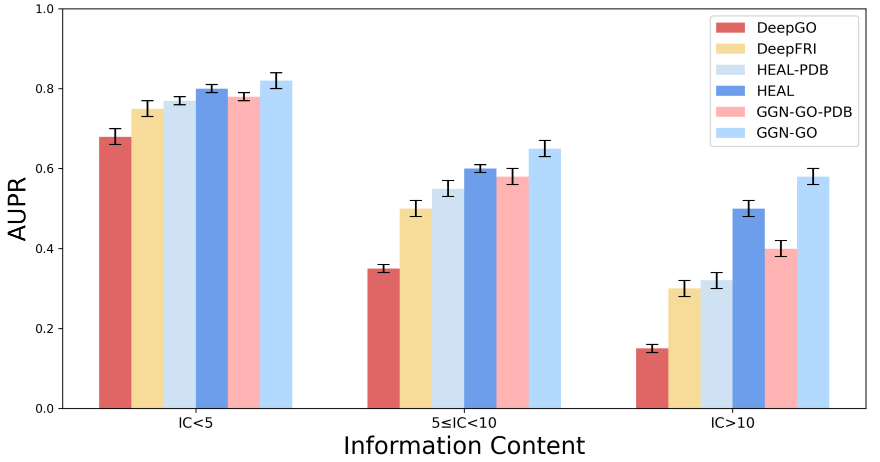

Figure 3: AUPR of different methods on PDBch test set over different IC (information content) thresholds.
